# Creating single Majorana type topological zero mode in superfluids of cold fermionic atoms

**DOI:** 10.1038/s41598-017-13641-4

**Published:** 2017-10-19

**Authors:** Xiao-Shan Ye, Yong-Jun Liu, Xiu Yun Zhang, Guoqing Wu

**Affiliations:** 1 College of Physics Science and Technology, Yangzhou University, Yangzhou, 225002 China; 2 Department of Physics and Astronomy, University of California, Los Angeles, California, 90095 USA

## Abstract

We explore the topological phase, which involves Majorana type topological zero mode fermions (MTZFs) at the edge, using *d*–wave superfluid with Rashba spin-orbit coupling (SOC) interactions. The self-Hermitian of this MTZF(${{\boldsymbol{\gamma }}}^{{\boldsymbol{\dagger }}}{\boldsymbol{(}}{\bf{k}}{\boldsymbol{)}}{\boldsymbol{=}}{\boldsymbol{\gamma }}{\boldsymbol{(}}{\bf{k}}{\boldsymbol{)}}$) is similar to that of the Majorana fermions (MFs) (${{\boldsymbol{\gamma }}}^{{\boldsymbol{\dagger }}}={\boldsymbol{\gamma }}$). We show that, to realize a single MTZF at each edge in superfluid with d–wave pairing in a Majorana type Kramers Doublet (MTKD) state, it is important to lift both the spin and the Dirac Cones degeneracies. These non-Abelian anyons obey the non-Abelian statistics which may be useful to realize topological quantum computation. We suggest that the topological feature could be tested experimentally in superfluids of cold fermionic atoms with laser field induced spin orbit interactions. These studies give a new possible way to investigate the MTZFs in a two-dimensional (2D) system as compared to MFs in the one-dimensional (1D) nano-wire and 2D system, and enrich the theoretical research on finding non-Abelian anyons in topological system.

## Introduction

Recently, the physics of characterizing topological phases of matter is intriguing intensely theoretical and experimental interests in condensed matter physics. One of the goals is to realize topological superconducting phases in real materials due to the possibility of finding MFs in these phases^1–12^. A MF is a fermion which only has half degree of freedom compared to the usual Dirac fermions. Due to its self-Hermitian(${\gamma }^{\dagger }=\gamma$), the MF is just an antiparticle of itself. These MFs play a critical role in realizing topological quantum computer because they may be utilized as a quantum qubit for their non-Abelian statistics^13–15^. So there have been considerable efforts to explore MFs for theoretical and application interests.

Several physical systems are suggested to be the candidates to find MFs, such as fractional quantum Hall states at filling factor $\nu =\mathrm{5/2}$ and $\mathrm{12/5}$ ^16,17^, a 2D system consisted of a topological insulator in proximity to a ferromagnet and a singlet superconductor^18^. To detect MFs, recently, it was also suggested that MFs can be found in superconducting vortices in a *s*–wave superconductor when it is sandwiched with a magnetic insulator and a thin semiconducting film^4,19^. With a strong SOC, some groups even suggest constructing MFs in quantum wires when they are located in proximity to superconductors in a magnetic field^5–7,20–22^. However, all these proposals are extremely challenging in experiments.

There are also some advices on generating MFs in chiral triplet superconductors^1,14,23^. In the case of spinless $p+ip$– wave state, one zero energy Majorana mode is obtained in a vortex. We usually call it a non-Abelian anyon, which supports non-Abelian statistics. Kitaev also suggests a 1D spinless toy model which supports Majorana zero modes at the ends of the *p*–wave superconducting chain^13^. However, in the spinful case in reality, there are two Majorana fermions at the edges, which form a usual complex fermion. So the non-Abelian statistics are not realized for this situation. To produce separated single MF, Ivanov *et al*.^14^ suggested using a half quantum vortex to suppress one of the two MFs in a vortex core in 2D systems. They show that this kind of vortice is non-Abelian anyon. Fujimoto^24^ advised applying magnetic field to generate single MF: a Zeeman field eliminates one MF and survives one MF associated with the Dirac cone in a vortex core. We may ask a question: can we find other methods to produce this kind of topological zero mode which is also a non-Abelian anyon?

Thanks to the great progress in ultra-cold atomic systems which have proven to be a powerful tool to simulate many quantum physics problems. In this article, we propose a scheme in which a single MTZF state, appearing at the edge of the superfluid system, is realized in cold fermionic atoms with laser field induced SOC interactions. Some previous studies focused on producing MFs in the *p*–wave pairing or *s*–wave pairing state^25^. However, the gaps of *p*–wave pairing are very small, and it is difficult to realize *p*–wave pairing using Feshbach resonance. Some suggest topological phases based on *s*–wave pairing superfluids of cold atoms, deemed to the realization of the MF states. This scenario needs a condition of a large magnetic field ($h > {\rm{\Delta }}$), which may destroy the pairing state by the orbital depairing effect. We point out that a topological superfluid (TS) can be realized in a d–wave superfluid with Rashba SOC interaction. We explore extensively the properties of topological phases in a *d*–wave superfluid. We show that a MTKD appearing at each edge of the TS. A MTKD is a pair of MTZFs localized at one edge of the lattices which is protected by a time-reversal symmetry (TRS). To realize MTKD in superfluids with *d*–wave pairing, we find that it is important to lift the Dirac Cones degeneracy. Interestingly, with increasing the strength of SOC, the MTKD is separated into two MTZFs in space: a single MTZF appears in a special zone at each edge of the TS. The single MTZF is very important for the non-Abelian statistics. So we provide a new way to generate the non-Abelian anyons. We notice that there are many papers study the Majorana physics in *d* wave superconductor. Some use different lattice geometry with *d* wave pairing^26–28^. Some focus on the topological property of different *d* wave pairing^29^. In this paper, we concentrate on the condition for creation of single MF in a topological MTKD state. There are two main parts of this paper. The first one is to demonstrate the picture of the topological phase for the case of spin-singlet pairing states. We consider the ${d}_{{x}^{2}-{y}^{2}}$ and ${d}_{{x}^{2}-{y}^{2}}+{d}_{xy}$–wave pairing case. In the second part of this paper, we give a detail analysis of the Majorana type zero mode edge states of the topological phase found in this model.

## Results

**Figure 1:**
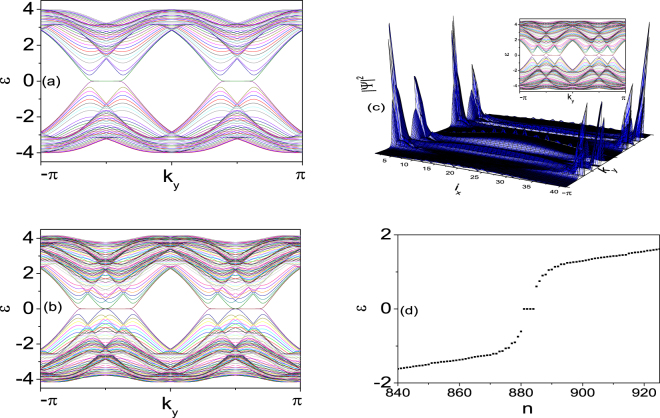
Energy spectra for systems with open boundaries for the *x*–direction and the periodic boundary condition for the *y*–direction. $\mu =0$, $h=0$, ${{\rm{\Delta }}}_{{d}_{{x}^{2}-{y}^{2}}}=1$, ${{\rm{\Delta }}}_{{d}_{xy}}=0.5$ and ${\alpha }_{L}=0.1$ (**a**), ${\alpha }_{L}=0.5$ (**b**). (**c**) Energy spectra and amplitude of the ground state wave functions of the zero energy mode for ${\alpha }_{L}=0.7$. (**d**)Quasiparticle excitation spectra for $h=0.5$ in the vortex lattice. The index numbers the eigenvalues.

Recently, some experiments demonstrating the synthetic SOC interaction in ultracold Fermi gases have stimulate a lot of interest in researching topological properties of ultracold atoms^30,31^. We note that the creation of *d*–wave Feshbach resonance is observed in low-entropy quantum degenerate gas of polar molecules in a 3*D* optical lattice^32,33^. Inspired by these experimental progresses, we explore a spin-singlet *d*–wave pairing superfluid with the Rashba SOC interaction in a 2D optical lattice in this paper. Using a tight-binding Hamiltonian in the 2D optical lattice, we consider a pairing interaction *V* between two elections on the nearest neighbor sites for ${d}_{{x}^{2}-{y}^{2}}$–wave and the next nearest neighbor sites for ${d}_{xy}$–wave pairing. The Hamiltonian is $H={H}_{kin}+{H}_{SOC}+{H}_{sc}$:

1 $$\begin{array}{rcl}\,\,{H}_{kin} & = & -\sum _{(ij)\sigma }t{c}_{i\sigma }^{\dagger }{c}_{j\sigma }-\sum _{{(ij)}^{\text{'}}\sigma }{t}^{\text{'}}{c}_{i\sigma }^{\dagger }{c}_{j\sigma }-\sum _{i\sigma }\mu {c}_{i\sigma }^{\dagger }{c}_{i\sigma }-h\sum _{i}({c}_{i\uparrow }^{+}{c}_{i\uparrow }-{c}_{i\downarrow }^{+}{c}_{i\downarrow }),\\ {H}_{SOC} & = & -{\alpha }_{L}\sum _{i}[({c}_{i-\hat{x}\downarrow }^{\dagger }{c}_{i\uparrow }-{c}_{i+\hat{x}\downarrow }^{\dagger }{c}_{i\uparrow })+i({c}_{i-\hat{y}\downarrow }^{\dagger }{c}_{i\uparrow }-{c}_{i+\hat{y}\downarrow }^{\dagger }{c}_{i\uparrow })+H\mathrm{.}c\mathrm{.],}\\ \,\,\,{H}_{sc} & = & \sum _{ij}[{{\rm{\Delta }}}_{ij}{c}_{i\uparrow }^{\dagger }{c}_{j\downarrow }^{\dagger }+h\mathrm{.}c\mathrm{.],}\end{array}$$

where ${c}_{i\sigma }^{\dagger }$ is the creation operator of a quasiparticle with spin $\sigma$ on site $i$, $t$ is the nearest neighbor hopping matrix element in the lattice plane, $t^{\prime}$ is that along the diagonal of each plaquette, $\mu$ is the chemical potential, and $h$ is the Zeeman field strength. The d–wave spin-singlet order parameter ${{\rm{\Delta }}}_{ij}=\frac{V}{2}\langle {c}_{i\uparrow }{c}_{j\downarrow }-{c}_{i\downarrow }{c}_{j\uparrow }\rangle$. The Rashba SOC strength is ${\alpha }_{L}$. Let us first study the edge states for the 2D ${d}_{{x}^{2}-{y}^{2}}+{d}_{xy}$–wave $({{\rm{\Delta }}}_{{d}_{{x}^{2}-{y}^{2}}}(\cos \,{k}_{x}-\,\cos \,{k}_{y})+{{\rm{\Delta }}}_{{d}_{xy}}\,\sin \,{k}_{x}\,\sin \,{k}_{y})$ superfluid in the absence of magnetic field. We impose the periodic boundary condition in the *y*– direction, and let the system have two open boundary edges in the *x*– direction. By solving the energy spectrum as a function of the momentum ${k}_{y}$, in Fig. 1, we illustrate the energy spectrum for the system with open boundary edges at ${L}_{x}=0$ and ${L}_{x}=41$. In this case, the edge zero modes appear. In the following, we will show that the edge zero modes are chiral MTZFs. These edge zero modes usually appear as the system has particle-hole symmetry of the Hamiltonian. We also find that the gapless edge states are doubly degenerated. According to Kramers theorem, we expect that these two Majorana type edge states form a Kramers pair. When we increase ${\alpha }_{L}$, we find the spin degeneracy is lifted. A single MTZF appears at some special regions of each edge of the TS. The energy spectrum of $H(k)$ with $h=0$ and ${\alpha }_{L}=0.5$ is shown in Fig. 1(b). The amplitudes of the ground-state wave functions are shown in Fig. 1(c) to confirm that the zero energy modes are edge states. If we change the chemical potential $\mu$ and the magnetic field $h$, we also find the MTZFs appear for ${k}_{y}\sim 0$ in the case of the ${d}_{{x}^{2}-{y}^{2}}({d}_{xy})$–wave pairing. We find out that these gapless edge modes are robust when the condition $-4t-\mu  < h < -\mu$ is satisfied. This result is consistent with the results discussed in reference^29^ for MFs. The existence of these zero energy states is not dependent on what kind of boundary the model is taken. To show this property, we have studied the vortex-lattice states using the same parameters in Eq. (1) with $h=0.5$ for the opening of bulk gap. The eigenvalues of the vortex-lattice states may be obtained by solving the Bogoliubov-de Gennes equations(Eq. (4) in the discussion). In Fig. 1(d) we show the quasiparticle spectra in the vortex-lattice system. As is seen, four zero energy eigenvalues are obtained by the numerical results. In fact, as we will discuss in the following, these zero energy states are protected by the particle-hole symmetry.

To understand the topological origin of the MTKDs, the Hamiltonian is rewritten in momentum space as

2 $$H=\frac{1}{2}\,\,\sum _{k}\,{{\rm{\Psi }}}_{k}^{+}H(k){{\rm{\Psi }}}_{k}\mathrm{.}$$

Here, ${\Psi }_{k}=({c}_{k\uparrow }{c}_{k\downarrow }{c}_{-k\uparrow }^{+}{c}_{-k\downarrow }^{+})$. $H(k)$ is given by

3 $$H(k)=(\begin{array}{cc}\varepsilon (k)-h{\sigma }_{z}+{H}_{soc} & i{{\rm{\Delta }}}_{k}{\sigma }_{y}\\ -i{{\rm{\Delta }}}_{k}^{\ast }{\sigma }_{y} & -\varepsilon (k)+h{\sigma }_{z}+{H}_{soc}^{\ast }\end{array}),$$

where the $\varepsilon (k)=-2t(\cos \,{k}_{x}+\,\cos \,\,{k}_{y})-\mu$. The Rashba SOC term is written as ${H}_{soc}={\alpha }_{L}(\sin \,{k}_{y}{\sigma }_{x}-\,\sin \,{k}_{x}{\sigma }_{y})$, where $\sigma {(\tau )}_{i}(i=\mathrm{0,}\,x,\,y,\,z)$ are the Pauli matrices.

When $h=0$, we explore the symmetry of the $H(k)$ using time reversal operator ${\rm{T}}$ $({\rm{T}}=i{\sigma }_{y}{\tau }_{0}{\rm{K}})$ and particle-hole operator ${\rm{P}}$ $({\rm{P}}={\sigma }_{0}{\tau }_{x}{\rm{K}})$, where ${\rm{K}}$ is the complex conjugation operator. It is easy to see that $H(k)$ have time reversal-invariant (${\rm{T}}H(k){{\rm{T}}}^{-1}=H(-k)$) and particle-hole symmetry (${\rm{P}}H(k){{\rm{P}}}^{-1}=-H(-k)$). We define an eigenvector $u$ by $H({k}_{y})u=Eu$. Due to the particle-hole symmetry, then we have $H({k}_{y})({\rm{P}}u)=-E({\rm{P}}u)$. ${E}_{n}\to -{E}_{n}$ and we have $u\to Pu={v}^{\ast }(-k)$. Considering the Bogoliubov quasiparticle operator is constructed in the usual way as ${\gamma }^{+}(k)={u}_{1}(k){c}_{\uparrow }^{+}(k)+{u}_{2}(k){c}_{\downarrow }^{+}(k)+{v}_{1}(k){c}_{\uparrow }(-k)+{v}_{2}(k){c}_{\downarrow }(-k)$, we immediately have ${\gamma }^{+}(k)=\gamma (k)$ if $u(k)$ is a zero mode of the Eq. (3). This implies the generation of MTZFs. Here, we introduce a winding number to characterize the topological properties of the system. Based upon the time reversal operator ${\rm{T}}$ and the particle-hole operator ${\rm{P}}$, we introduce the operator ${\rm{\Gamma }}=-i{\rm{PT}}$, which anticommutes with $H(k)$: ${[H(k),{\rm{\Gamma }}]}_{+}=0$. Using the basis $$U=\frac{1}{\sqrt{2}}(\begin{array}{ll}{{\bf{I}}}_{\mathrm{2\ast 2}} & {\sigma }_{y}\\ {\sigma }_{y} & {{\bf{I}}}_{\mathrm{2\ast 2}}\end{array})$$ which diagonalizes ${\rm{\Gamma }}$: $${U}^{\dagger }\Gamma U=(\begin{array}{ll}{{\bf{I}}}_{\mathrm{2\ast 2}} & 0\\ 0 & -{{\bf{I}}}_{\mathrm{2\ast 2}}\end{array})$$, we off-diagonalizes the Hamiltonian: $${U}^{\dagger }H(k)U=(\begin{array}{ll}0 & {\bf{A}}\\ {{\bf{A}}}^{\dagger } & 0\end{array})$$, where ${\bf{A}}=-(\varepsilon (k)-{\sigma }_{z}+{H}_{soc})+i{\rm{\Delta }}(k){\sigma }_{y}$. The topological phase of the system can be characterized by the winding number:

4 $$\begin{array}{rcl}{W}_{N}({k}_{y}) & = & \frac{1}{4\pi i}{\int }_{-\pi }^{\pi }d{k}_{x}tr[{A}^{-1}(k){\partial }_{{k}_{x}}A(k)-{A}^{\dagger -1}(k){\partial }_{{k}_{x}}{A}^{\dagger }(k)]\\  & = & -\frac{1}{2\pi i}{\int }_{-\pi }^{\pi }d{k}_{x}tr[A(k){\partial }_{{k}_{x}}A{(k)}^{-1}]\\  & = & \frac{1}{2\pi i}{\int }_{-\pi }^{\pi }d{k}_{x}ln[detA(k\mathrm{)].}\end{array}$$

The winding number is ${W}_{N}=0$ at ${k}_{y}=0$ and around ${k}_{y}=0.5\pi$(modulus of 2) when ${\alpha }_{L} < 0.5$. On the other hand, for ${\alpha }_{L} > 0.5$, the winding number is ${W}_{N}=1$ around ${k}_{y}=0.5\pi$. The existence of winding number implies the existence of topological order in the system. In general, the change of the winding number indicates both Abelian topological order and non-Abelian topological order emergence. There are not any non-Abelian anyons in Abelian topological order. However, the non-Abelian topological order obeys the non-Abelian statistics which are essential to construct decoherence-free qubits. Here, we use $\nu ={(-\mathrm{1)}}^{{W}_{N}}$ as a hallmark to show when the non-Abelian topological phase appears. When $\nu =1$, there is an Abelian topological phase in the system. In the present case, $\nu =1$ indicates the appearance of MTKD at each edge of the TS. When $\nu =-1$, there is a single MTZF displays at each edge of the TS. However, we should remind that, when $h=0$, time-reversal symmetry holds, according to the Kramers theorem, there are two pair of zero energy bound states in our system. As we stressed in our paper, we only separate the two pair of zero energy bound states in space when we introduce the SOC interaction. So we can find a single MTZF appears at some zones of the edge in the superfluid when the system is under the open edge condition. These zones have $\nu =-1$.

**Figure 2:**
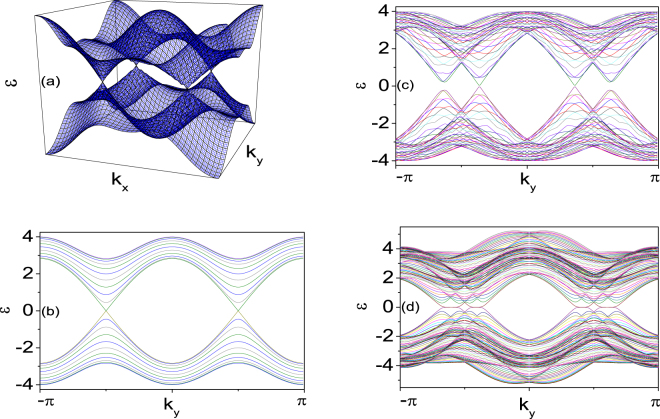
Energy spectra for systems with periodic boundary condition for the *x*– and *y*–direction. $\mu =0$, $h=0$, ${{\rm{\Delta }}}_{{d}_{{x}^{2}-{y}^{2}}}=1$, ${{\rm{\Delta }}}_{{d}_{xy}}=0$ and ${\alpha }_{L}=0$ (**a**). Energy spectra for system with open boundaries for *x*–direction (**b**). Energy spectra for system with open boundaries for *x*–direction and the periodic boundary condition for the *y*–direction. $\mu =0$, $h=0$, ${{\rm{\Delta }}}_{{d}_{{x}^{2}-{y}^{2}}}=1$, ${{\rm{\Delta }}}_{{d}_{xy}}=0$ and ${\alpha }_{L}=0.5$ (**c**). Energy spectra for system with open boundaries for *x*–direction and the periodic boundary condition for the *y*–direction. $\mu =0$, $h=0$, ${{\rm{\Delta }}}_{{d}_{{x}^{2}-{y}^{2}}}=1$, $t^{\prime} =0.5$ and ${\alpha }_{L}=0.5$ (**d**).

To realize MTKD or a single MTZF at each edge in TS with a *d*–wave pairing, we find that it is important to lift both the spin and Dirac Cones degeneracy. In Fig. 2, we show the energy spectrum of the system when we introduce the ${d}_{xy}$-wave pairing. We find that the ${d}_{xy}$-wave lifts the Dirac Cones degeneracy which is the usual case in ${d}_{{x}^{2}-{y}^{2}}$-wave superconductors. When we introduce the SOC interaction, the spin degeneracy is also lifted. By increasing the SOC interaction strength, we find that the MTKD or a single MTZF appears at each edge in the superfluid when the system is under the open edge condition. But, only lifting the spin degeneracy cannot realize MTKD or a single MTZF at each edge in the *d*–wave superfluid (Fig. 2(c)). Inspired by this result, we also consider to lift the Dirac Cones degeneracy with the next nearest neighbor hopping matrix. In Fig. 2(d), we show that the edge zero modes in the ${d}_{{x}^{2}-{y}^{2}}$-wave superfluid appear when we consider the one diagonal next nearest neighbor hopping matrix $t^{\prime} =0.5$. We can see that the next nearest neighbor hopping matrix $t^{\prime}$ lifts the Dirac Cones degeneracy (Fig. 2(d)). When we introduce the SOC interaction, the spin degeneracy is also lifted. The zero modes appear at the edges of some regions. This is also an important way to realize a single MTZF at each edge from the MTKD edge states.

## Discussions

To better understand the present results, we also give a few remarks in the following. First, we map our d–wave superfluid Hamiltonian into the widely known spin-triplet chiral *p*–wave superconductor model. This mapping is helpful to intuitively understand the origin of the topological order presented above. In the Hamiltonian, the normal dispersion $\varepsilon (k)=\varepsilon (k)+{H}_{SOC}$ can be diagonalized as $\varepsilon (k)=\varepsilon (k)+{\sigma }_{z}{\varepsilon }_{1}(k)$ using the chirality basis $O(k)$, where ${\varepsilon }_{1}(k)=det({H}_{SOC})$ and

$$O(k)=\frac{1}{\sqrt{2}|{\varepsilon }_{1}(k)|}(\begin{array}{cc}{\alpha }_{L}\,{\sin }\,{k}_{y}-i{\alpha }_{L}\,{\sin }\,{k}_{x} & -{\varepsilon }_{1}(k)\\ {\varepsilon }_{1}(k) & {\alpha }_{L}\,{\sin }\,{k}_{y}+i{\alpha }_{L}\,{\sin }\,{k}_{x}\end{array}).$$

With this chirality basis, the hamiltonian $H(k)$ can be given by

$${\mathbb{H}}(k)=(\begin{array}{cc}\varepsilon (k)+{\varepsilon }_{1}(k){\sigma }_{z} & i{\rm{\Delta }}(k)[{O}^{\dagger }{\sigma }_{y}{O}^{\ast }(-k)]\\ -i{{\rm{\Delta }}}^{\ast }(k)[{O}^{T}(-k){\sigma }_{y}O(k)] & \varepsilon (k)-{\varepsilon }_{1}(k){\sigma }_{z}\end{array})$$

with the following unitary transformation:

$$H(k)={(\begin{array}{cc}{O}^{\dagger }(k) & 0\\ 0 & {O}^{T}(-k)\end{array})}^{\dagger }{\mathbb{H}}(k)(\begin{array}{cc}{O}^{\dagger }(k) & 0\\ 0 & {O}^{T}(-k)\end{array}).$$

We define

$$\tilde{{\rm{\Delta }}}(k)=i{\rm{\Delta }}(k)[{O}^{\dagger }{\sigma }_{y}{O}^{\ast }(-k)]=(\begin{array}{cc}({\alpha }_{L}\,{\sin }\,{k}_{y}+i{\alpha }_{L}\,{\sin }\,{k}_{x})\frac{{\rm{\Delta }}(k)}{{\varepsilon }_{1}(k)} & 0\\ 0 & ({\alpha }_{L}\,{\sin }\,{k}_{y}-i{\alpha }_{L}\,{\sin }\,{k}_{x})\frac{{\rm{\Delta }}(k)}{{\varepsilon }_{1}(k)}\end{array})$$

**Figure 3:**
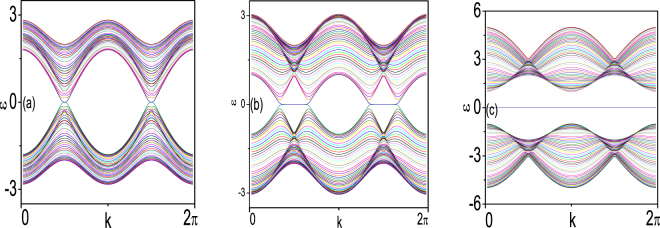
Energy spectra for a *p*–wave system with open boundaries for the *x*–direction. $\mu =0$, $h=0$, ${{\rm{\Delta }}}_{p}=0.1$ (**a**), ${{\rm{\Delta }}}_{p}=0.5$ (**b**), ${{\rm{\Delta }}}_{p}=1.5$ (**c**).

Thus we can write the paring matrix

$$\tilde{{\rm{\Delta }}}=(\begin{array}{cc}{d}_{x}+i{d}_{y} & 0\\ 0 & {d}_{x}-i{d}_{y}\end{array}),$$

where ${d}_{x}=({\tilde{{\rm{\Delta }}}}_{\downarrow \downarrow }-{\tilde{{\rm{\Delta }}}}_{\uparrow \uparrow }\mathrm{)/2}$ and ${d}_{y}=-i({\tilde{{\rm{\Delta }}}}_{\downarrow \downarrow }+{\tilde{{\rm{\Delta }}}}_{\uparrow \uparrow }\mathrm{)/2}$. This indicates that the topological property of our system is similar to that of spin triplet chiral $p+ip$– wave superconductor. To demonstrate the importance of creating and lifting the Dirac Cones degeneracy in order to create the multi-MTZFs at the edge of the system, we show how the multi-MTZFs can be created at the edge of the system by lifting the Dirac Cones degeneracy in the well-known 1D spinless *p*–wave superconductor model. This degeneracy lifting can drive the system into a different phase. A typical model of the 1D *p*–wave superconductor with modulated chemical potentials is given by $H=\,\,{\sum }_{i}\,(t{c}_{i}^{+}{c}_{i+1}+{\rm{\Delta }}{c}_{i}{c}_{i+1}+{\mu }_{i}{c}_{i}^{+}{c}_{i})$ where ${\mu }_{i}=V\,\cos \,\mathrm{(2}\pi i\alpha +{k}_{\delta })$ with $V$ being the strength and $\alpha$ a rational number^34^. This model also can be viewed as Kitaev ladder topological superconductors with $\mu ^{\prime} =\mu +{t}_{y}\,\cos \,\frac{n}{N+1}\pi$, where ${t}_{y}$ is the interchain transfer integral, $n$ indicates the site along the chain and $N$ is the number of the Kitaev chains^35^. We show the spectra under open edge condition at a typical examples of $\alpha =\mathrm{1/2}$ in Fig. 3, We can see that under open edge condition, two zero modes appear in the bulk gap for the whole phase parameter space when pairing potential ${\rm{\Delta }}$(${\rm{\Delta }}=0.1$) is small. As ${\rm{\Delta }}$ increases (${\rm{\Delta }}=0.5$), the Dirac Cones degeneracy is lifted, and four Dirac Cones are created. We can see that zero mode Majorana type solutions only exist in two lifted degeneracy Dirac Cone zones, and they cannot appear in the zone of separated Dirac points (Fig. 3(b)). As ${\rm{\Delta }}$ is increased further (${\rm{\Delta }}=1.5$), the zero modes in the bulk gap exist in the whole parameter space. This corresponds to a topological phase transition. The phase diagram can be referenced in^35^ with different number of the Kitaev chains.

**Figure 4:**
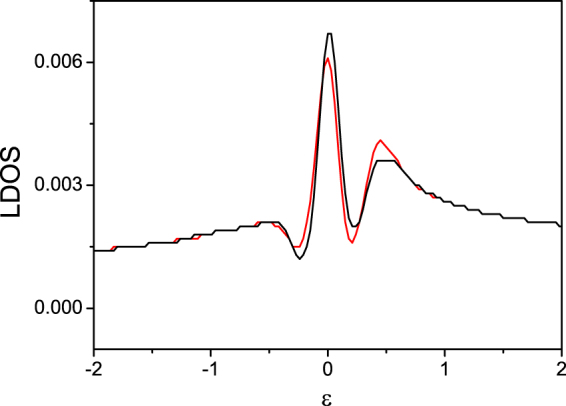
Local Density of States (LDOS) of the spin selective Andreev reflection at the center of the vortex for the BdG calculations. The LDOS for spin-up $N(\uparrow )(E)$(red) and spin-down $N(\downarrow )(E)$(black) are different.

To detect the Majorana type zero models (MTZMs) suggested in this paper, we will show that the non-Abelian anyons in a vortex give a spin selective reflection spectrum. Taking into account of this property, we can detect the non-Abelian anyons in a vortex core using spin density profiles. First, in experiment, creating vortices in the SOC system is essential to realize the non-Abelian anyons. We suggest the lasers which generate the SOC interaction should be replaced by those carrying orbital angular momentums. The spatially separated vortices can be generated with the help of this kind of laser. This setup can be referred to^3^. Second, we notice that He *et al*.^36^ have pointed out that spin selective Andreev reflections(SSAR) are induced by MTZM at the end of a nanowire: an Andreev reflection will happen with electrons in the same spin of the MTZM, while not to happen in electrons with opposite spins. This novel Andreev reflection between the same spin electrons is different from the ordinary Andreev reflection. This property may serve to reveal the existence of the MTZMs. So, our suggestion is that we could detect this kind of SSAR at the center of the vortex. To show this SSAR spectrum theoretically, we calculate the local density of state (LDOS) using the eigenfunction of the system Hamiltonian. The energy eigenvalues and eigenfunction may be obtained by solving the Bogoliubov-de Gennes equations:

5 $$\sum _{j}\,(\begin{array}{cc}{H}_{ij,\sigma } & {{\rm{\Delta }}}_{ij}\\ {{\rm{\Delta }}}_{ij}^{\ast } & -{H}_{ij,\bar{\sigma }}^{\ast }\end{array}){{\rm{\Psi }}}_{j}={E}_{n}{{\rm{\Psi }}}_{i},$$

where ${\rm{\Psi }}$ can be written as ${{\rm{\Psi }}}_{j}^{T}=({u}_{j,\uparrow }^{n},{u}_{j,\downarrow }^{n},{v}_{j,\uparrow }^{n},{v}_{j,\downarrow }^{n})$. Here ${u}^{n}$ and ${v}^{n}$ are the Bogoliubov quasiparticle with corresponding eigenvalues ${E}_{n}$. With the energy eigenvalues and eigenfunction, the LDOS is given by

6 $$\begin{array}{rcl}\rho ({{\bf{r}}}_{i},E) & = & -\,\frac{1}{{M}_{x}{M}_{y}}\sum _{n,\sigma }\,[|{u}_{i\uparrow }^{n}{|}^{2}f^{\prime} ({E}^{n}-E)\\  &  & +\,|{v}_{i\downarrow }^{n}{|}^{2}f^{\prime} ({E}^{n}+E)]\end{array}$$

where $f^{\prime} (E)$ is the derivative of the Fermi distribution function. For the calculation we use the unit cell of size ${M}_{x}\times {M}_{y}=41a\times 41a$. We notice that the node exciting of the gap nodes may be merged into zero energy mode exciting spectrum, and thus, it is difficult to identify the MTZMs. To avoid node exciting, we add a *s*–wave pairing. Though *s*–wave pairing with SOC interaction may give MTZMs, a large magnetic field satisfying $h > {{\rm{\Delta }}}_{s}$ is required to get the topological order. So, the zero energy exciting in the vortex core is due to the MTZMs of the *d*–wave topological order. The calculation results are shown in Fig. 4. In the figure, we observe spin-polarization dependence of the zero bias differential tunneling conductance at the center of the vortex, which may be ascribed to the spin selective Andreev reflection. This result is consistent with the experimental results show by Jia *et al*.^37^ in spin polarized scanning tunneling microscope (STM) experiments. The experimental dictation of the MTZMs can also use charge-impurity-induced Majorana fermions in topological superconductors because of a pair of MFs bounded at the two sides of one charge impurity and well separated. The corresponding local density of states is explored by the authors^38^.

The experimental realization and dictation of the MTZMs can also be realized in real superconducting compounds. To get ${d}_{{x}^{2}-{y}^{2}}+{d}_{xy}$–wave pairing superconductors, one can grow quasi-one dimensional nanowires along the direction tilted with angle $\theta$ with respect to the *x*–axis where it shows ${d}_{{x}^{2}-{y}^{2}}$–wave pairing as suggested in ref.^28^. In fact, as we pointed, we can realize the MTZMs by increasing the one diagonal of the next nearest neighbor hopping matrix in a square lattice which shows ${d}_{{x}^{2}-{y}^{2}}$–wave pairing. This unidirectional neighbor hopping can be achieved by increasing the pressure on the superconductors. One can have this kind of superconductor though the chemical pressure using a chemical substitution. So, we suggest that the organic superconductor $\kappa -{(ET)}_{2}X$ is a suitable candidate^39,40^. In this compound, it is believed that the superconducting paring symmetry is ${d}_{{x}^{2}-{y}^{2}}$–wave. Using a different iron *X* substitution, which corresponds to give a chemical pressure, we can easily change the diagonal next nearest neighbor hopping matrix in a square lattice.

## Summary

In summary, we explore the MTKD and single MTZF in *d*–wave superfluids. We point out that TS can be realized in a *d*–wave superfluid with the Rashba SOC interaction. We show that a MTKD appears at each edge of the TS. To realize MTKD in TS with a *d*–wave pairing, we find that it is important to lift the spin and Dirac Cones degeneracies. Interestingly, with the increase of the strength of SOC, a single MTZF appears at each edge of the TS. The single MTZF is very important on non-Abelian statistics, so we give a new way to generate the non-Abelian anyons. We also give some suggestions on the realization and dictation the MTZFs in *d*–wave superfluids.
